# Protease Substrate-Independent Universal Assay for Monitoring Digestion of Native Unmodified Proteins

**DOI:** 10.3390/ijms22126362

**Published:** 2021-06-14

**Authors:** Emmiliisa Vuorinen, Salla Valtonen, Nazia Hassan, Randa Mahran, Huda Habib, Morteza Malakoutikhah, Kari Kopra, Harri Härmä

**Affiliations:** 1 Department of Chemistry, Chemistry of Drug Development, University of Turku, Vatselankatu 2, 20500 Turku, Finland; samaval@utu.fi (S.V.); nazia.n.hassan@utu.fi (N.H.); randa.r.mahran@utu.fi (R.M.); huda.n.habib@utu.fi (H.H.); morteza.malakoutikhah@utu.fi (M.M.); khkopr@utu.fi (K.K.); harri.harma@utu.fi (H.H.); 2 Department of Virology, Institute of Biomedicine, University of Turku, Kiinamyllynkatu 13, 20520 Turku, Finland

**Keywords:** protease activity, protease inhibition, digestion, time-resolved luminescence, label-free

## Abstract

Proteases are a group of enzymes with a catalytic function to hydrolyze peptide bonds of proteins. Proteases regulate the activity, signaling mechanism, fate, and localization of many proteins, and their dysregulation is associated with various pathological conditions. Proteases have been identified as biomarkers and potential therapeutic targets for multiple diseases, such as acquired immunodeficiency syndrome, cardiovascular diseases, osteoporosis, type 2 diabetes, and cancer, where they are essential to disease progression. Thus, protease inhibitors and inhibitor-like molecules are interesting drug candidates. To study proteases and their substrates and inhibitors, simple, rapid, and sensitive protease activity assays are needed. Existing fluorescence-based assays enable protease monitoring in a high-throughput compatible microtiter plate format, but the methods often rely on either molecular labeling or synthetic protease targets that only mimic the hydrolysis site of the true target proteins. Here, we present a homogenous, label-free, and time-resolved luminescence utilizing the protein-probe method to assay proteases with native and denatured substrates at nanomolar sensitivity. The developed protein-probe method is not restricted to any single protein or protein target class, enabling digestion and substrate fragmentation studies with the natural unmodified substrate proteins. The versatility of the assay for studying protease targets was shown by monitoring the digestion of a substrate panel with different proteases. These results indicate that the protein-probe method not only monitors the protease activity and inhibition, but also studies the substrate specificity of individual proteases.

## 1. Introduction

Proteases are vital enzymes that modify proteins by cutting them at specific digestion sites. This is achieved by hydrolyzing a peptide bond either between two amino acids in the middle of the peptide chain (endopeptidase) or at the terminus of the peptide (exopeptidases). Enzymes are grouped based on their catalytic mechanism, and in humans, these protease groups are aspartic, cysteine, serine, threonine, glutamic acid, and metalloproteases. Even within a single protease group, the substrate specificity can vary significantly [1]. This sequence preference leads to differences in enzyme specificity as the longer the target sequence is, the more specific the protease typically is [2]. This highlights the fact that proteases even within one group digest a variety of substrates, [1,3] and thus, it is complex to develop an universal assay to monitor the activity of multiple proteases [4].

Proteases control multiple processes in the human body, such as digestion, cell cycle, wound healing, and immune system activation [1,5,6]. They also play a part in multiple diseases such as, the human immunodeficiency virus protease in acquired immunodeficiency syndrome [7], peptidyl dipeptidase A (ACE) in cardiovascular diseases [8], cathepsin K in osteoporosis [9,10], β-site APP-cleaving enzyme in Alzheimer’s disease [11,12], serine aminopeptidases in type 2 diabetes [13], and various proteases in cancers [14,15]. Multiple protease inhibitors have been developed and are currently under investigation for the treatment of these diseases such as ACE inhibitors for hypertension medication [16,17]. To perform protease studies effectively, there is a growing need for protease assays that are more sensitive, rapid, and easy to use. 

Traditionally, protease activity is studied by separating the digested substrate fragments with e.g., by using liquid chromatography [18]. Thereafter, the purified peptide fragments are further quantified using mass spectrometry [19]. These methods are time-consuming and require specialized, expensive instrumentation. They are also unsuitable for high-throughput screening (HTS). Thus, homogeneous assay methods based on absorbance or fluorescence have become popular [4,20].

Universal HTS compatible protease activity methods using various assay dedicated substrates have been developed based on absorbance and fluorescence detection. The methods often require labeling of the substrate protein or peptide. Azocasein and casein are the most common target proteins used in these digestion monitoring techniques. Azocasein functions by releasing the azo-dye for absorbance detection. Casein operates by releasing aromatic amino acid residues which react with Folin’s reagent to form chromophoric products [21,22]. Quantification methods are based on fluorescence, Förster resonance energy transfer (FRET), and bioluminescence resonance energy transfer (BRET) detection which typically utilize fluorescein isothiocyanate-labeled casein. Additionally various FRET and BRET pair labelled peptide structures containing specific enzyme substrate sequences are utilized [23,24]. Peptides are the most commonly used substrates for FRET and BRET studies, as peptides can be readily labeled in situ [20,25,26,27,28]. Moreover, fluorescence polarization (FP) has been utilized for protease activity monitoring. In FP, the rotational changes of a fluorescently labeled substrate protein or large peptide are monitored as a function of enzyme digestion [29,30].

Although the absorbance and fluorescence methods are widely used today, labeling of proteins and peptides makes the methods difficult to control, cumbersome to prepare, and costly. As the methods typically utilize labelled substrates or peptide sequences instead of natural substrate proteins of the individual protease of interest, substrate selection or labeling may prevent digestion causing false results or non-functional assay. To address these issues, we have developed a label-free protease assay utilizing an external Eu-probe. The protein-probe assay is an end-point type mix-and-measure method, which has been previously demonstrated for the detection of protein–ligand and protein–protein interactions [31,32]. In the context of protease activity monitoring, the main advantage of the protein-probe technique is the substrate-independent detection of a wide range of non-modified non-labeled intact substrate proteins. The Eu-probe is not present at the time of digestion, and thus the potential interferences can be avoided. Here, we demonstrate the universal applicability and functionality of the protein-probe method with several different substrate proteins, and by using three model proteases.

## 2. Materials and Methods

### 2.1. Materials, Instrumentation, and Assay Buffers

The 9-denate Eu^3+^-chelate, {2,2′,2″,2‴-{[4′-(4‴-isothiocyanatophenyl)-2,2′,6′,2″-terpyridine-6,6″-diyl]bis(methylene-nitrilo)}tetrakis(acetate)}europium(III) was obtained from QRET Technologies (Turku, Finland). The chelate was conjugated according to the manufacturer’s instructions to the Eu-probe peptide (H_2_N-EYEEEEEVEEEVEEE) (Pepmic Co., Ltd., Suzhou, China). The Eu-probe was purified as described before, [31] and the concentration was determined using the DELFIA technique and a commercial EuCl_3_ standard from PerkinElmer Life and Analytical Sciences, Wallac (Turku, Finland). The protein substrates, recombinant pertussis toxin (PTX), G protein alpha I subunit (Gαi) [33], human Ras GTPase-activating protein 1 (p120GAP) [34], wild type Kirsten RAt Sarcoma virus (KRAS, 2-188), human son of sevenless SOS1 catalytic domain (SOS^cat^, 564–1048) [35], and eukaryotic initiation factor 4A1 (eIF4A1) [32] were kind gifts from our collaborators. Malate dehydrogenase (MDH) was purchased from Roche (Basel, Switzerland). All other reagents, including carbonic anhydrase (CA), bovine serum albumin (BSA), pronase, pepsin, papain, Pepstatin A, and E-64 were purchased from Sigma-Aldrich (St. Louis, MO, USA). Thermal ramping assays were performed on black Framestar 96-well PCR plates (4titude, Surrey, UK) and all the other assays were performed on black OptiPlate 384-well microtiter plates from PerkinElmer (Groeningen, Netherlands).

The Eu-probe was purified using reverse phase liquid chromatography, Dionex ultimate 3000 LC system from Dionex Corporation (Sunnyvale, CA, USA) and Ascentis RP-amide C18 column from Sigma-Aldrich (St. Louis, MO, USA) [31,32]. Time-resolved luminescence (TRL) measurements were performed with Tecan Spark 20M from Tecan Life Sciences (Männedorf, Switzerland), using 340 nm excitation wavelength, 620 nm excitation wavelength, 400 µs integration time, and 800 µs delay time. A PTC-100 Programmable Thermal Controller from MJ Research, Inc. (Watertown, MA, USA) was used for thermal ramping.

All assays were performed in 8 µL protease reaction volume, followed by 65 µL detection solution added after the enzyme reaction. Unless otherwise specified, the pronase, papain and pepsin assay reactions were performed in Pronase Buffer (10 mM HEPES, pH 7.5, 0.001% (*m*/*v*) Triton X-100), Papain Buffer (10 mM HEPES, pH 7.5, 0.001% (*w*/*v*) Triton X-100, 5 mM cysteine), and Pepsin Buffer (pH 3; 4.1 mM Na_2_HPO_4_, 6.1 mM citric acid, pH 3, 0.001% (*w*/*v*) Triton X-100), respectively. In all cases, the end-point detection was performed using the protein-probe detection solution (pH 4; 7.7 mM Na_2_HPO_4_, 7.9 mM citric acid, 0.01% (*w*/*v*) Triton X-100, 3.5 µM 1,1,3,3,3′,3′-hexamethylindodicarbocyanine iodide (HIDC), 1 nM Eu-probe). 

### 2.2. Potease Activity and Inhibition Monitoring Utilizing the Protein-Probe Technique

Pronase (0–6.4 µM), pepsin (0–0.1 µM), and papain (0–16 µM) were titrated with intact BSA (10 nM), and intact CA (100 nM) and heat denatured CA (3 nM) as substrates. For all molar concentration calculations with pronase protease cocktail, the estimated average molecular weight of 50,000 g/mol was used. The heat denaturation of CA was performed before protease assay by heating 90 nM CA in MilliQ water for 3 min at 75 °C. Digestion reaction was performed by incubated protease and substrate for 30 min at 37 °C (8 µL). After digestion, the reaction was cooled down to RT (room temperature for 5 min) and the protein-probe solution containing 1 nM Eu-probe was added in 65 µL volume. Result for the end-point protease activity assay was monitored using TRL-signal detection at RT (5 min).

Inhibitor titrations were performed with pepsin (25 nM) and papain (3 nM), using Pepstatin A (0–500 nM) and E-64 (0–400 nM) as inhibitors, respectively. The protease and inhibitor were first incubated for 5 min, prior to the addition of native (100 nM) or heat denatured (3 nM) CA used as a substrate. Digestion was performed as previously using 30 min incubation at 37 °C. After digestion, the end-point protein-probe addition and TRL-signal monitoring were performed as previously. 

### 2.3. Universal, Substrate Independent Protease Activity Monitoring with the Protein-Probe

The thermal curves for the substrate proteins, eIF4A1 (150 nM), PTX (100 nM), MDH (100 nM), SOS^cat^ (10 nM), BSA (3 nM), CA (50 nM), KRAS (50 nM), Gαi (100 nM), p120GAP (20 nM), were monitored by heating the sample from 35 to 90 °C in 5 °C intervals following the protocol introduced previously [31,32]. Samples were heated for 3 min at each temperature in 8 µL reaction volume using HEPES-based assay buffer (10 mM HEPES, 0.001% (*w*/*v*) Triton X-100, 1 mM MgCl_2_). Heating was followed by the addition of 65 µL protein-probe solution, and TRL-signals were monitored after 5 min at RT. 

Nine different native substrate proteins (eIF4A1, PTX, MDH, SOS^cat^, BSA, CA, KRAS, Gαi, p120GAP) used at two concentrations (25 and 50 nM) were digested with pronase and papain (12.5, 25, 50, and 100 nM). The protease–substrate mixtures were incubated for 10 min at 37 °C to enable digestion. This step was followed by the direct detection of the native substrate by using protein-probe or post-digestion heat denaturation of substrates followed by the protein-probe detection as previously mentioned. The post-digestion heat denaturation was performed by heating the substrate protein for 3 min at 60 °C (p120GAP, PTX, MDH, eIF4A1, SOS^cat^, Gαi, and BSA) or 70 °C (KRAS, and CA).

### 2.4. Data Analysis

The S/B ratio was calculated as µ_max_/µ_min_ and coefficient of variation (CV%) as (σ/µ) × 100. In these formulas, µ is the mean value and σ is the standard deviation (SD). The denaturation temperatures (T_m_), half-maximal inhibitory concentration (IC_50_), and half-maximal effectivity concentration (EC_50_) were obtained using standard sigmoidal fitting functions in Origin 2016 (OriginLab, Northampton, MA, USA).

## 3. Results and Discussion

**Figure 1:**
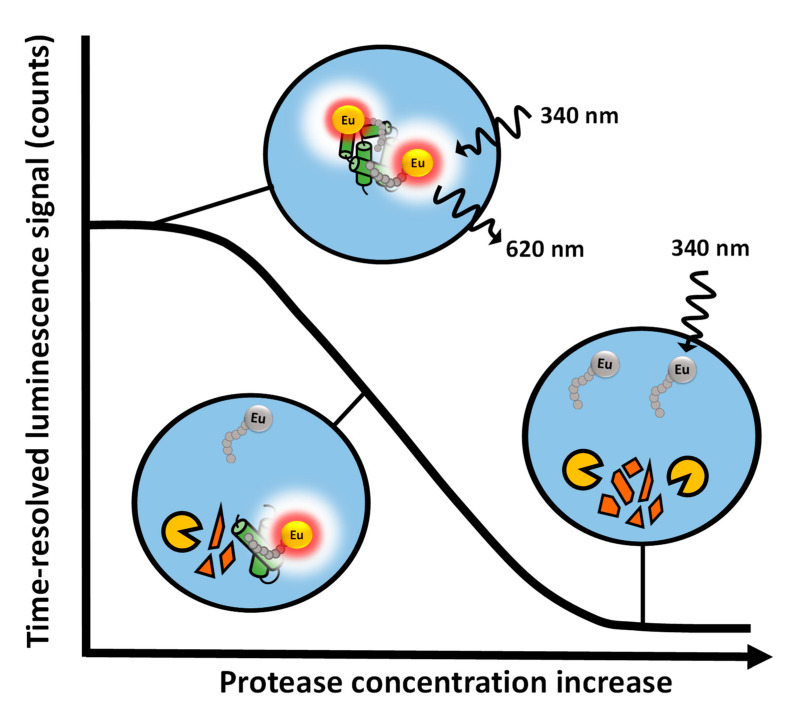
The principle of the protein-probe method for monitoring substrate digestion and protease activity. When no protease is present or it is inhibited, the Eu-probe can interact with the substrate protein, leading to a high TRL-signal in the modulation solution. Substrate digestion by active protease prevents the Eu-probe binding to the digested substrate. Thus the Eu-probe protection is lost, and low TRL-signal is monitored.

Proteases are a large group of enzymes that have been identified as biomarkers and potential drug targets for several diseases [7,8,9,10,11,12,13,14,15]. However, at the same time proteases are a very heterogeneous group of enzymes, with varying digestion site requirements. Thus, it can be challenging to study different proteases with a single method. We have previously developed a label-free protein-probe method for studying stability and interactions of proteins [31,32]. The protein-probe requires no labeling of the studied proteins, as an external Eu-probe is utilized in an end-point measurement. Thus, the protease and substrate protein remain intact and require no label or additional tags enabling the fulfillment of criteria for a label-free assay technique. Now, we present the method for monitoring protease activity using several proteases and a panel of substrate proteins. The novelty of the protein-probe lies in substrate independency, which no other homogenous method in the market provides without separate labelling of each individual substrate protein. The protein-probe method is based on the measurement of the Eu-probe TRL-signal in an end-point fashion. The substrate digestion reaction is initially performed in a small volume prior to the addition of Eu-probe in modulation solution i.e., the protein-probe. When the Eu-probe is bound to a non-digested substrate protein, high TRL-signal is monitored, in comparison to the low TRL-signal upon functional digestion of the target substrate (Figure 1).

### 3.1. The Protein-Probe Monitors Protease Activity with High Sensitivity

**Figure 2:**
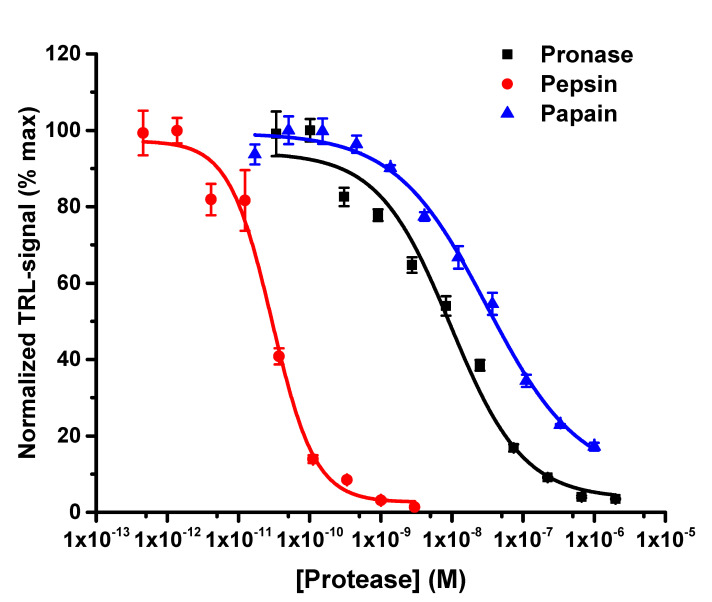
Protease titrations with BSA as the digested substrate. Pronase (black, 0–2 µM), pepsin (red, 0–3 nM), and papain (blue, 0–1 µM) were mixed with 10 nM BSA, and reactions were incubated at 37 °C for 30 min. Thereafter the protein-probe solution was added, and the TRL-signals were monitored at RT. The EC_50_ values for pronase, pepsin, and papain were 8.6 ± 2.7, 0.03 ± 0.01, and 31.0 ± 8.5 nM, respectively. The S/B ratios of non-digested and digested BSA were 11.1, 21.1, and 4.8 for pronase, pepsin, and papain, respectively. Data represent mean ± SD (*n* = 3).

The protein-probe was developed as a substrate-independent protease activity tool, and to study the effect of both substrate and protease concentration. We first performed a protease titration with three model proteases, pronase (0–2 µM) (a protease cocktail from the extracellular fluid of *Streptomyces griseus*), pepsin (0–3 nM), and papain (0–1 µM) to digest BSA (10 nM). Protease reactions were performed in protease specific buffer by using 30 min incubation at 37 °C. This was performed before the addition of the protein-probe solution, enabling the unaffected digestion of the substrate and end-point detection of the reaction. This digestion protocol was selected instead of optimizing the temperature and time for each protease reaction separately. The only optimizations were made to ensure protease activity, and thus 5 mM cysteine for papain and pH 3 buffer for pepsin were selected (data not shown). The protein-probe monitored the digestion of BSA with all three proteases, giving the EC_50_ values of 8.6 ± 2.7, 0.03 ± 0.01, and 31.0 ± 8.5 nM for pronase, pepsin, and papain, respectively (Figure 2). S/B ratios calculated from non-digested and digested BSA were 11.1, 21.1, and 4.8 for pronase, pepsin, and papain, respectively. These results indicate that the protein-probe can efficiently monitor the digestion of BSA in a protease concentration-dependent manner, using low and sub nM protease substrate.

**Figure 3:**
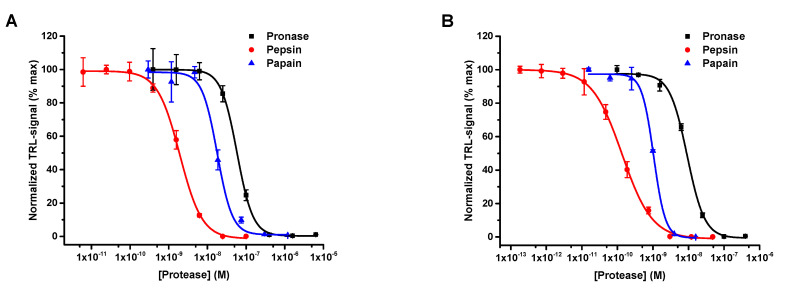
Comparison of native and heat-denatured CA as a substrate for proteases. (**A**) Native CA (100 nM) was used as a digested substrate for pronase (black), pepsin (red), and papain (blue). The EC_50_ values were 58.3 ± 1.0, 1.9 ± 0.1, and 16.8 ± 2.0 nM, with the respective S/B ratios from 34 to 114. (**B**) Pronase (black), pepsin (red), and papain (blue) were similarly titrated with heat-denatured CA (3 nM) as the protease substrate. The EC_50_ values of 9.2 ± 0.6, 0.14 ± 0.01, and 1.0 ± 0.05 nM were obtained, with S/B ratios from 62 to 117, respectively. In all assays, protease reactions were first incubated for 30 min at 37 °C, before end-point detection using the protein-probe and respective TRL-signal monitoring. Data represent mean ± SD (*n* = 3).

After successful BSA digestion, the protease activity was studied with CA as a substrate. Based on our previous thermal shift assays, heating of CA and other selected proteins increases the monitored TRL-signal, due to protein denaturation [31]. Thus we next investigated the effect of thermal denaturation of the substrate protein on the assay performance. Protein denaturation can affect the protease functionality by exposing the digestion sites or hiding them upon aggregation. Thus, both denatured and native form of the CA were studied as a substrate. Concentrations for CA testing were chosen to achieve similar S/B ratios with both native and heat-denatured CA (data not shown). Therefore, native (100 nM) and heat-denaturated (3 nM, 3 min at 75 °C) CA samples were compared in a similar protease titration as previously performed with BSA. The EC_50_ values detected for pronase, pepsin, and papain were 58.3 ± 1.0, 1.9 ± 0.1, and 16.8 ± 2.0 nM with the native CA, and 9.21 ± 0.62, 0.14 ± 0.01, and 1.05 ± 0.05 nM with the denatured CA, respectively (Figure 3). The S/B ratios with intact CA were 34, 114, and 59 and with denatured CA 89, 117, and 62 for pronase, pepsin, and papain, respectively. These results demonstrate that CA denaturation significantly reduces the need of both substrate and protease, maintaining the high S/B ratio. However, substrate concentration reduction has no linear effect on the digestion rate, as ΔEC_50_ varied from 6 to 16-fold depending on the protease. One obvious reason is the fact that denatured CA might have an open protein structure, which potentially is more digestible in comparison to the intact CA. Another explanation is related to the low substrate and protease concentrations potentially affecting the protease activity through substrate–enzyme affinity, as the equilibrium between the molecules is different. Nevertheless, the data prove that both intact and denatured proteins are equally applicable as protease substrates.

### 3.2. The Protein-Probe Method Enables Efficient Protease Concentration and Inhibition Dependend Activity Monitoring

**Figure 4:**
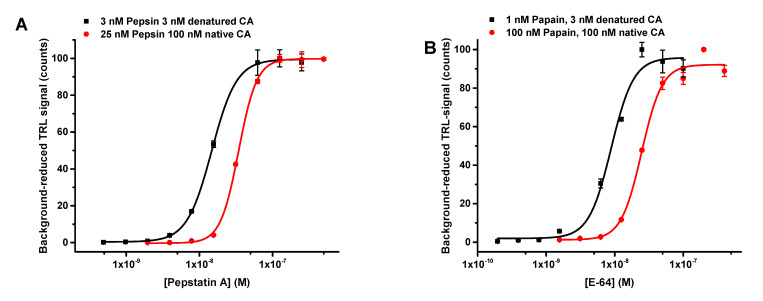
Inhibitor titrations with pepstatin A and E-64 for pepsin and papain with intact and denatured CA as a digested substrate. (**A**) Pepstatin A titration was performed using 25 nM pepsin for intact (100 nM, red) and 3 nM pepsin for denatured (3 nM, black) CA. The monitored IC_50_ values were 34.3 ± 0.5 nM and 14.7 ± 0.2 for intact and denatured CA, respectively. (**B**) E-64 inhibition was studied with 100 nM papain for intact (100 nM, red) and denatured (3 nM, black) CA. The monitored IC_50_ values were 24.7 ± 1.8 and 8.9 ± 0.9 nM for the native and denatured CA, respectively. With both inhibitors the EC_50_ values obtained using the protein-probe technique were similar to those reported previously. Data represent mean ± SD (*n* = 3).

Impaired protease activity often has negative effects on biological functions in humans and are linked to various diseases. Thus, multiple drug development programs targeting proteases and their inhibitors have been launched [16,17,36]. This led us to study the potential of the protein-probe technique for the monitoring of protease inhibitors. These tests were performed with two concentrations of pepsin (3 nM and 25 nM) and papain (1 nM and 100 nM). Pronase, a cocktail of several proteases, was not investigated, as this would have required a cocktail of inhibitors. We selected aspartic proteinase inhibitor pepstatin A (0–500 nM) for pepsin, and irreversible cysteine protease inhibitor E-64 (0–400 nM) for papain to be studied with intact (100 nM) and heat denatured (3 nM) CA. Proteases were incubated with their respective inhibitor for 5 min before substrate addition and further incubation for 30 min at 37 °C. For pepstatin A, the IC_50_ values monitored were 34.3 ± 0.5 nM and 14.7 ± 0.2 nM, and similarly for E-64 the values were 24.7 ± 1.8 and 8.9 ± 0.9 nM, when using the native or denatured CA, respectively (Figure 4). Both blockers are potent low nM inhibitors [37,38,39,40], and as expected, the IC_50_ values monitored with different protease concentrations were also different. More reliable and protease concentration-independent results were obtained with denatured CA, and with the low protease concentrations. The monitored values are also in a good agreement with the IC_50_ values reported previously, since IC_50_ values of 5–15 nM for pepstatin A, and the IC_50_ value of 9 nM for E-64/papain has been reported [37,38,39]. These findings indicate that the protein-probe is a highly potential method also for protease inhibitor testing.

### 3.3. The Protein-Probe Enables Universal, Substrate-Independent Protease Activity Monitoring

The heterogeneity of proteases and their substrates pose a problem for assay developers, as many available assays are not suitable for more than a single, assay-specific substrate, ruling out the studies on intact protease-specific substrates. The ultimate goal of the protein-probe assay was to enable the monitoring of multiple proteases in a substrate-protein-independent manner. Ability to use non-denatured protease-specific substrates provides a unique solution to measure the protease of interest with their natural target proteins, thus mimicking their function in a cellular context. This potentially shows that protease specificity patterns can also be studied.

To demonstrate the substrate-independent detection, digestion of nine different model substrates (eIF4A1, PTX, MDH, SOS^cat^, BSA, CA, KRAS, Gαi, and p120GAP) is carried out. These proteins have varying sizes and shapes, and thus the panel can model different types of substrate proteins of interest. To study the assay functionality in neutral buffer, pepsin was excluded from this test as the low pH required for pepsin activity denatures many of the chosen substrates (data not shown). Assays were performed at two substrate concentrations (25 or 50 nM), with pronase (0–100 nM) and papain (0–100 nM), using 10 min protease reaction time at 37 °C. Based on our previous knowledge, we anticipated that not all proteins are equally observed by the protein-probe method, and some proteins at low nM concentration are not detectable when intact. As shown previously [31] and with CA, denaturation increases the substrate visibility for the protein-probe. To provide digestion protocol utilizing intact proteins, we designed a protocol where the substrate is exposed to heat denaturation after digestion. For this, we monitored the T_m_ values for all used substrates (Table 1), and selected to denature substrates either at 60 or 70 °C for 3 min after the protease reaction. Assays with all substrates were also performed without this post-denaturation step to compare the results. Thus, we set two experiments side-by-side by performing the digestion and signal monitoring identically in both cases. The only difference was the thermal denaturation performed before the addition of the protein-probe solution.

**Figure 5:**
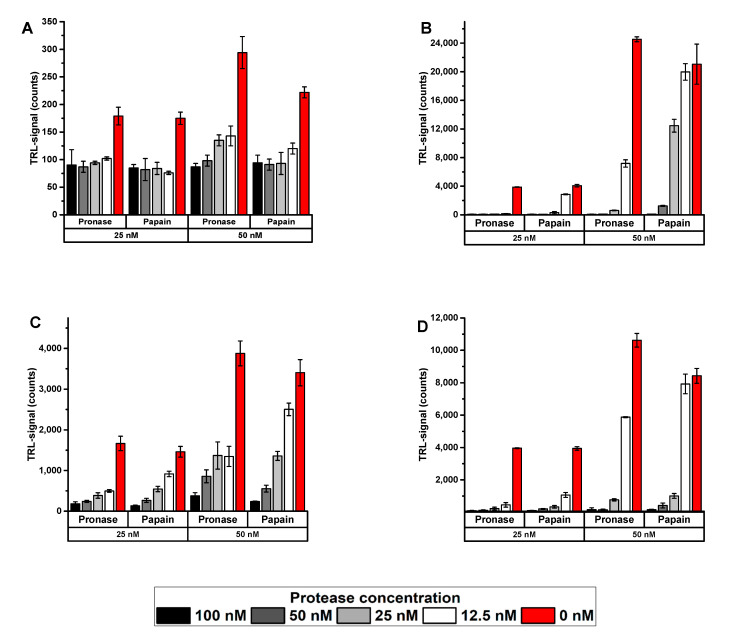
The digestion of MDH and eIF4A1 with pronase and papain. Pronase and papain (0–100 nM) were assayed with native MDH and eIF4A1 (25 or 50 nM) by using 10 min incubation at 37 °C. The detection was performed directly after digestion at RT (**A**,**C**) or using the substrate post-denaturation after the digestion (**B**,**D**) to yield increase in the TRL-signal and substrate detectability. MDH gave only low TRL-signal at RT, which was increased significantly upon heating-induced post-denaturation of the target substrate. For eIF4A, increased TRL-signals were monitored upon thermal post-denaturation, but the protein was already detectable in its native form, even at 25 nM concentration. Data represent mean ± SD (*n* = 3).

Using the protein-probe with the selected substrates, seven of the nine proteins gave a clear signal without heating at 50 nM concentration, whereas with 25 nM substrate concentration the number dropped to five (data not shown). After post-digestion heat denaturation, all tested substrates were measurable at both concentrations. With 50 nM substrate, the heat denaturation increased the maximal TRL-signal for four of the non-digested substrates from 5 to 95-folds over the signal obtained without denaturation. Some of the proteins gave high signal when intact, and heat denaturation had only minor TRL-signal-increasing effect. MDH, which was not measurable in its intact form, resulted in the highest signal increase upon denaturation with an approximately 100-fold increase of S/B ratio with both proteases (Table 2, Figure 5). The substrate denaturation after digestion had no effect on the measurability of the digestion efficiency, but only the detected signal level (Table 2). This extra heating step was especially beneficial for PTX, MDH, KRAS, and eIF4A1. All digestion efficiencies were calculated with or without heat denaturation from the background-reduced TRL-signals obtained with substrate and with and without the protease of interest. However, even the digestion efficiency was unchanged between the protocols, the digestion efficiency greatly varied when the proteases were compared among different substrates. Pronase efficiently digested many of the substrates and was most active with PTX and KRAS, while higher concentration of pronase was required for Gαi, BSA, and CA. Papain, on the other hand, was especially efficient with p120GAP and PTX, but less active with SOS^cat^ and BSA. This clearly proves the importance of substrate selection and shows the specificity of different proteases.

MDH and eIF4A1 were chosen to study the protein-probe functionality in more detail based on their detectability with and without post-digestion heat denaturation. At the given concentrations (25 or 50 nM), the MDH resulted in a very low TRL-signal at RT and a significant increase upon heating, while the eIF4A1 was highly detectable without heating and only modest increase upon heating occurs at the used conditions (Table 2, Figure 5). The heating of MDH increased the TRL-signals by more than 90-fold both in pronase and papain buffers (Figure 5A,B). In case of eIF4A1, heating also increased the measured TRL signal by approx. 2.6-fold in both buffers, although the heating was unnecessary due to the high signal level at RT. In case of both substrate proteins, the digestion efficiency was mainly affected by the protease concentration. The ability to use low substrate concentration improves the assay sensitivity and enables low protease concentrations, thus providing an inexpensive approach to investigate proteases and their substrates. Between these two tested concentrations, 50 nM substrate, unsurprisingly, yielded significantly higher signal levels with both MDH and eIF4A1, especially after heat denaturation. Compared to the 50 nM MDH the digestion efficiency of the lowest protease concentration increased with the 25 nM heat-denatured substrate up to 1.4- and 7.1-fold with pronase and papain, respectively. At higher protease concentrations this effect diminishes. This indicates that less protease is required for digestion of smaller substrate concentrations and this effect is enhanced by post-digestion denaturation, due to the increase detectability of the substrate.

With this panel of substrate proteins, we have shown that the protein-probe enables efficient protein digestion studies in a label-free format. The protein-probe assay is performed using a two-step protocol to separate the digestion reaction and the detection. This enables free and protease-dependent selection of buffer and avoids possible interferences as the Eu-probe is not present during the active protease reaction. The low pH used in protein-probe solution also terminates most of the protease reactions, which is beneficial to end-point reaction monitoring. However, some substrate proteins are not highly visible for the protein-probe and some proteases might cause unwanted background signal. All proteases used in this study, however, were non-visible for the protein-probe even after heat-denaturation (data not shown). On the other hand, substrate protein detectability could be increased by post-digestion denaturation. In this study, all selected proteases were expected to digest the target substrate at multiple point, increasing the method functionality, as these small fragments are undetectable. However, it remains to be studied how the function of other types of enzymes can be monitored using the protein-probe technique. 

## 4. Conclusions

Here, we have presented the protein-probe technique for monitoring the protease activity and inhibition with several different proteases and substrate proteins in a homogeneous and label-free format, utilizing TRL-signal detection. We have shown that various proteases can be investigated in a substrate-independent manner, using native non-denatured protein substrates. This essentially mimics natural protein digestion by proteolytic enzymes in a cellular context, providing substrate and inhibition specificity studies. The method enables substrate-focused studies at nanomolar protein concentrations without any labeling steps to investigate proteins and ligands. Unfortunately, some proteins were not highly detectable with the protein-probe at RT. Thus, we introduced a protocol to heat denature the substrate protein after digestion. This extended the potential of the protein-probe to study a greater variety of substrates, some of which are not highly detectable at RT in their native form at the used concentration level. This makes the protein-probe a versatile and a first-in-class truly universal method for proteases and their substrates.

## Figures and Tables

**Table 1:** Temperatures were selected for substrate screening based on the denaturation temperatures of these substrate proteins.

Substrate Protein	Tm (°C)	Temperature for Substrate Screening
p120GAP	53.6 ± 0.6	60 °C
PTX	54.1 ± 0.6	60 °C
KRAS	62.7 ± 0.3	70 °C
MDH	44.6 ± 0.5	60 °C
Gαi	56.9 ± 0.6	60 °C
eIF4A1	54.9 ± 0.2	60 °C
SOS^cat^	45.3 ± 0.2	60 °C
BSA	57.0 ± 1.3	60 °C
CA	67.9 ± 0.9	70 °C

**Table 2:** Digestion efficiency (%) of papain and pronase at different concentrations with variety of native substrates monitored with the protein-probe.

		Digestion Efficiency (%)				S/B Ratio (Sample/Buffer)			
		Pronase		Papain		Pronase		Papain	
Substrate	Protease (nM)	Native ^a^	Denatured ^b^	Native	Denatured	Native	Denatured	Native	Denatured
p120GAP	*100*	99	100	98	99	1	1	2	2
	*50*	95	98	98	99	4	3	2	2
	*25*	87	83	97	98	9	19	3	3
	*12.5*	78	68	97	100	14	35	3	1
	*0*	–	–	–	–	58	106	71	121
PTX	*100*	97	100	94	100	1	1	1	1
	*50*	93	99	87	100	1	2	2	1
	*25*	91	100	86	100	2	1	2	1
	*12.5*	87	99	77	100	2	1	2	1
	*0*	–	–	–	–	7	58	5	54
KRAS	*100*	95	100	88	95	1	1	2	2
	*50*	96	100	82	84	1	1	2	5
	*25*	93	99	66	55	2	2	2	11
	*12.5*	91	99	62	49	2	3	3	13
	*0*	–	–	–	–	9	168	5	24
MDH	*100*	88	100	ND ^c^	100	1	1	1	1
	*50*	82	100	ND	95	2	1	1	16
	*25*	65	98	ND	52	2	9	1	140
	*12.5*	62	72	ND	5	2	110	2	276
	*0*	–	–	–	–	5	385	2.9	291
Gαi	*100*	82	73	82	71	10	25	7	29
	*50*	71	61	79	73	15	35	8	27
	*25*	65	55	60	62	18	40	14	38
	*12.5*	61	45	57	62	20	50	15	38
	*0*	–	–	–	–	50	89	33	99
eIF4A1	*100*	96	99	92	99	3	2	5	2
	*50*	86	96	80	99	6	5	11	2
	*25*	62	89	66	93	16	13	17	10
	*12.5*	27	13	67	38	29	95	17	88
	*0*	–	–	–	–	39	110	48	142
SOS^cat^	*100*	96	99	86	57	5	3	19	77
	*50*	92	99	77	35	8	4	31	117
	*25*	89	98	63	26	11	7	49	133
	*12.5*	75	96	49	12	23	14	66	157
	*0*	–	–	–	–	90	327	130	180
BSA	*100*	79	84	79	78	26	18	20	14
	*50*	69	73	74	74	38	30	24	17
	*25*	52	59	65	68	59	44	31	21
	*12.5*	37	46	52	52	77	57	42	31
	*0*	–	–	–	–	121	106	88	62
CA	*100*	94	98	80	77	2	5	4	24
	*50*	89	48	72	57	3	106	5	44
	*25*	79	4	60	31	4	192	6	70
	*12.5*	65	10	42	14	7	181	8	87
	*0*	–	–	–	–	17	200	14	101

^a^ Non-denatured native sample. ^b^ Post-digestion heat denatured sample. ^c^ ND refers to less than three-fold TRL-signal increase obtained for the protein substrate compared to protein-probe in buffer, thus deemed to unreliable results.

## Data Availability

Not applicable.

## Abbreviations

ACE	peptidyl dipeptidase A
BRET	Bioluminescence energy transfer
BSA	Bovine serum albumin
CA	Carbonic anhydrase
CV	Coefficient of variation
EC_50_	Half-maximal effectivity concentration
eIF4A1	Eukaryotic translation initiation factor 4A1
FP	Fluorescence polarization
FRET	Förster resonance energy transfer
Gαi	G protein alpha I subunit
HIV	Human immunodeficiency virus
HTS	High-throughput screening
IC_50_	Half-maximal inhibitory concentration
KRAS	Kirsten RAt Sarcoma virus
MDH	Malate dehydrogenase
PTX	Pertussis toxin
p120GAP	Human Ras GTPase-activating protein 1
RT	Room temperature
SD	Standard deviation
SOS^cat^	Son of sevenless catalytic domain
S/B	Signal to background
T_m_	Denaturation temperature
TRL	Time-resolved luminescence
